# Rationally Designed Oral DOX Gels for Colon-Specific Administration

**DOI:** 10.3390/gels8120759

**Published:** 2022-11-22

**Authors:** Jie Li, Luping Ma, Cheng Wang, Pengju Jiang, Pengfei Cui, Jianhao Wang

**Affiliations:** School of Pharmacy, Changzhou University, Changzhou 213164, China

**Keywords:** pectin, DOX, chalk, oral

## Abstract

Colorectal cancer (CRC) is the third leading cause of death from cancer in both men and women. Traditional CRC dosage forms deliver the drug to both desired and unwanted sites of drug action, resulting in a number of negative side effects. Chemotherapeutic and chemopreventive agents are being targeted and delivered directly to the colon and rectum using targeted oral drug delivery systems. The main challenge in successfully targeting drugs to the colon via the oral route is avoiding drug absorption/degradation in the stomach and small intestine before the dosage form reaches the colon. In this study, we employed biocompatible chalk to adsorb DOX, then mixed pectin and cross-linked with calcium ions to form PC–DOX gels. The presence of cross-linked pectin and chalk can provide dual protection for the drug, significantly reducing drug leakage in gastric acid. In vitro release results showed that the designed PC–DOX could achieve 68% colon delivery efficiency. In the simulated colon environment, the released semi-degradable chalk did not affect the uptake of doxorubicin by colon cancer cells. Finally, in vivo simulation experiments in mice showed that rationally designed PC–DOX could achieve the highest colonic delivery efficiency. Our strategy has great potential for application in the treatment of colon cancer.

## 1. Introduction

Over the last decade, colorectal cancer has been ranked as the third most common cancer in the world, as well as one of the leading causes of cancer-related deaths [1]. With localized colon cancer, the five-year survival rate is 90%, but it drops to 14% in patients with metastases to distant organs [2]. Surgical resection is the mainstay of treatment for primary colon cancer [3]. The most commonly used chemotherapies, such as 5-fluorouracil (5-FU), platinum derivatives, and folic acid (FA), are typically administered intravenously [4,5,6]. Various literature suggests that adherence to oral administration is the highest in the evaluation of different routes of administration. However, only a few chemotherapy drugs have been proven and used in clinical practice, and these chemotherapy drugs are capecitabine, which is an orally administered prodrug of 5-FU, and tegafur-uracil [7,8,9]. These drugs need to overcome the following limitations: First, the stability of the drug is poor in harsh stomach conditions. Second, the first-pass effect of the liver leads to low bioavailability of the drug. Finally, the mucus barrier prevents the penetration and subsequent absorption of the drug. Overcoming these limitations can improve the therapeutic effect of oral preparations for colorectal cancer [10,11].

Doxorubicin hydrochloride (DOX) is a water-soluble anticancer drug that belongs to the first generation of anthracyclines. It is used to treat different types of cancer [12]. DOX inserts into DNA and stops the replication process, leading to cell death. Although doxorubicin is effective, its administration may cause some adverse side effects including rapid clearance, liver accumulation, accumulation, and irreversible cardiomyopathy. To eliminate or reduce these side effects, different administration methods have been proposed, including the use of different drug delivery systems (DDS) [13,14,15]. The application of DDS can also promote the prolonged release of DOX, thereby reducing the possibility of overdose. Pectin, as a plant-derived polysaccharide, has been applied to colon-specific administration [16]. Pectin cannot be digested by the mammalian stomach and small intestine, but colon bacteria can secrete pectinase, and when the drug-coated pectin is orally transport to the colon, it can be degraded by pectinase, releasing drugs and enabling colon-targeted drug delivery. However, oral delivery needs to go through the stomach acid environment, and the drug will be leaked. In order to improve the colon-targeting effect and reduce the leakage in the stomach and small intestine, researchers chose high acetyl substituted pectin against gastric acid permeability. In addition, due to the presence of a large number of carboxyl groups in pectin molecules, calcium ions can also be added to form a gel to increase strength [17].

Calcium-carbonate-based material plays an important role in drug delivery due to its good flexibility [18]. Chalk is a form of biocompatible limestone composed of the mineral calcite and originally formed deep under the sea by the compression of microscopic plankton that had settled to the sea floor [19,20]. The porous structure of chalk endows it with strong adsorption capacity [21].

**Scheme 1:**
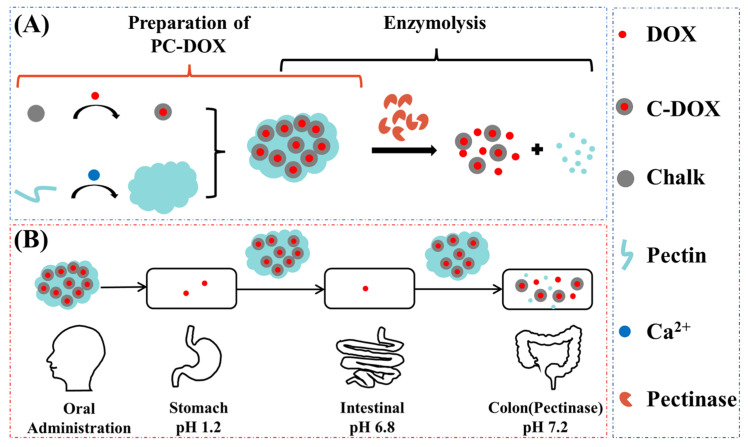
(**A**) Preparation and enzymolysis of PC–DOX; (**B**) the process of PC–DOX oral administration.

In this study, we employed biocompatible chalk to adsorb DOX, then mixed pectin and cross-linked with calcium ions to form a PC–DOX jelly. Calcium ions can mediate the pectin solution to form a gel to prevent drug leakage [22]. Chalk is a biocompatible natural material with porous structure that has been widely used in the field of medicine. The presence of cross-linked pectin and chalk can provide dual protection for the drug, significantly reducing drug leakage in gastric acid. When the delivery system reaches the colon, in the presence of colonic enzymes secreted by bacteria, pectin is degraded, resulting in a local gastric acid environment, accelerating the release of drugs, and achieving colon-specific administration. In vitro release results showed that the designed PC–DOX could achieve 68% colon delivery efficiency. In the simulated colon environment, the released semi-degradable chalk did not affect the uptake of doxorubicin by colon cancer cells. Finally, in vivo simulation experiments in mice showed that rationally designed PC–DOX could achieve the highest colonic delivery efficiency (Scheme 1). Our strategy has great potential for application in the treatment of colon cancer.

## 2. Results and Discussion

### 2.1. Preparation and Characterization of Pectin-Chalk DOX Gels (PC–DOX)

**Figure 1:**
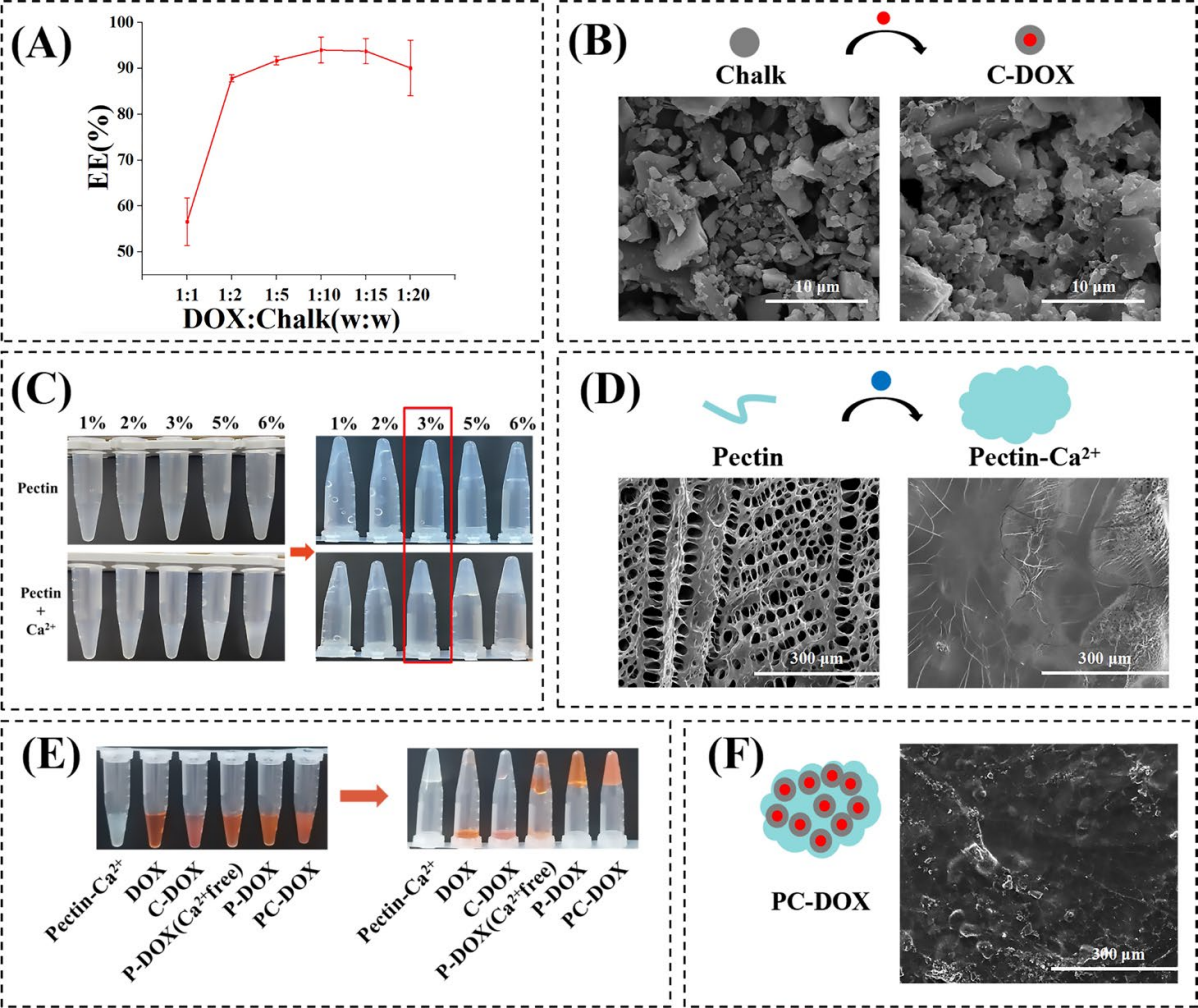
Preparation and characterization of pectin-chalk DOX gels (PC–DOX). (**A**) The encapsulation efficiency of DOX; (**B**) SEM of chalk and C–DOX; (**C**) different concentrations of pectin and pectin-Ca^2+^ gels; (**D**) SEM of pectin and pectin-Ca^2+^ gels; (**E**) all control and experimental groups of PC–DOX; (**F**) SEM of PC–DOX.

An important factor affecting the efficiency of colon-specific drug delivery is the impact of the digestive tract, especially the direct impact of gastric acid. In this study, the cross-linking of pectin and the adsorption of chalk added two lines of defense to prevent the leakage of drugs in the stomach. First, we mixed chalk with DOX to evaluate the drug adsorption efficiency of chalk (C–DOX). The results showed that when the concentration of DOX was determined, the adsorption amount of DOX increased with the increase of the amount of chalk and reached the peak at 1:10 (DOX:Chalk, *w*/*w*), so this ratio was used for subsequent research (Figure 1A). SEM results showed that the morphology of chalk did not change significantly before and after adsorption of DOX (Figure 1B). Next, we tested the conditions under which pectin formed gels. Pectin can be dissolved in water, and CaCl_2_ was added to form a gel. As shown in Figure 1C, we tested the effects of pectin concentration on gelation, and finally chose 3% (Figure 1D). Then, we mixed C–DOX with the pectin solution and added CaCl_2_ to test its final morphology. It was found that a red gel was finally formed (Figure 1E). SEM results also showed that C–DOX was uniformly dispersed in pectin (Figure 1F). At the same time, we also prepared other groups as controls.

### 2.2. DOX Release Study of PC–DOX

**Figure 2:**
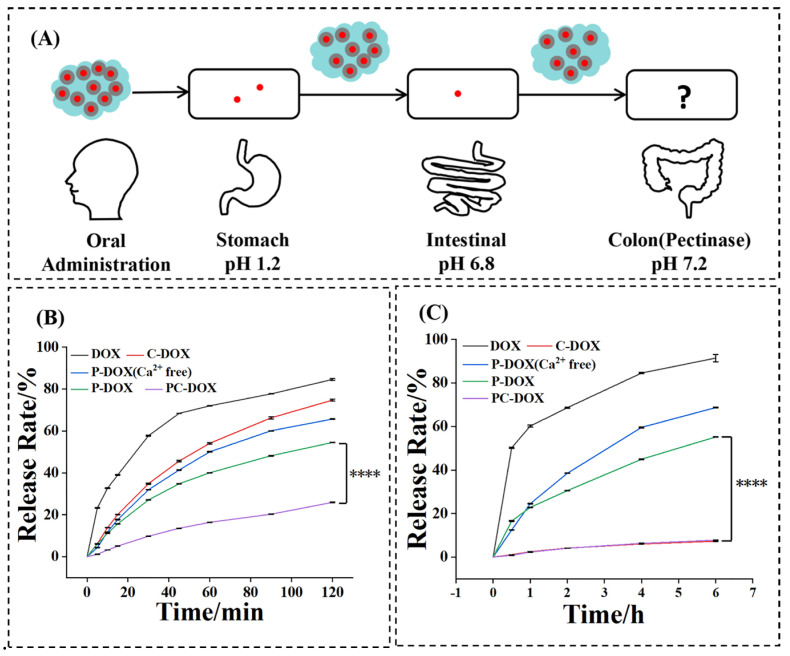
(**A**) Schematic diagram of the oral administration of PC–DOX; (**B**) the diagram of simulating drug release in the stomach (pH 1.2) **** *p* < 0.0001 (*n* = 3); (**C**) the diagram of simulating drug release in the intestine (pH 6.8) **** *p* < 0.0001 (*n* = 3).

To test drug release in the stomach and small intestine, we performed simulated gastric juice and simulated intestinal fluid drug release experiments (Figure 2A). The gastric emptying time of food was 2–4 h, and the 2 h assay results showed that the release of PC–DOX was the lowest in the simulated gastric environment at pH 1.2, with only 17% DOX leakage (Figure 2B). With only chalk adsorption, 80% of C–DOX was released, while 50% of P–DOX without chalk adsorption was released. If only pectin and DOX were mixed, the release of the P–DOX (Ca-free) group was more obvious. In short, the adsorption of chalk, the protective effect of pectin, and the cross-linking effect of Ca are indispensable in preventing the release of drugs in gastric acid. This result proves the rationality of our designed PC–DOX. When the oral drug is emptied from the stomach, it enters the small intestine. The retention time in the small intestine is about 6 h, and the pH in the small intestine is about 6.8. The release results showed that the PC–DOX group and the C–DOX group were almost not released in the small intestine (Figure 2C), while other groups had a higher proportion of release. After passing through the stomach and small intestine, the drug enters the colon. Through the above two releases, we performed calculations. As Table 1 shows below, about 68% of DOX in the PC–DOX group could reach the colon, while much less of other groups could. This indicates that the materials we prepared may have a good application prospect.

### 2.3. Enzyme-Responsive Release and Cell Uptake Assay

**Figure 3:**
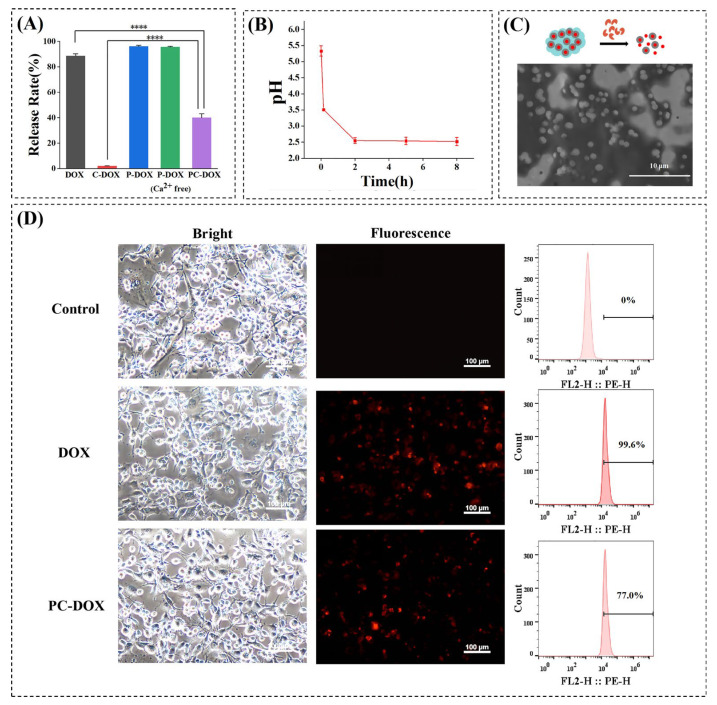
(**A**) Drug-release rates of PC–DOX and other control groups after the addition of pectinase *** *p* < 0.001, **** *p* < 0.0001 (*n* = 3); (**B**) the pH curve of the enzymatic hydrolysis process; (**C**) SEM of PC–DOX that was enzymatically hydrolyzed; (**D**) images about cell uptake and flow cytometry to quantify the fluorescence intensity in cells.

The pH in the colon is about 7.2, and the bacteria in the colon can secrete pectinase. In order to simulate the release of PC–DOX in the colon, we configured a pH 7.2 pectinase solution as a release medium and incubated with different groups of preparations for 8 h. The results showed that (Figure 3A) there was almost no release in the C–DOX group, which was in line with our expectations, because chalk did not respond to the above conditions. We noticed that most of the drugs in P–DOX and P–DOX (Ca-free) groups were released, which was related to the enzymatic hydrolysis of pectinase. Although the drugs in P–DOX and P–DOX (Ca^2+^-free) can be released in the colon, most of them had been released in the stomach and small intestine (Figure 2, Table 1), and little could reach the colon site. Surprisingly, in the PC–DOX group, which was rarely released in the stomach and small intestine, less than 40% of the drug was released in the colon. Referring to the release of C–DOX, it can be speculated that the adsorption of DOX by chalk inhibited the release of the drug, but the design of this project required PC–DOX to be released in the colon and taken up by the target cells. Therefore, we freeze-dried the digested sample and performed electron microscopy. The results showed that compared with that in the PC–DOX (Figure 1F) before digestion, after pectinase digestion, the size of the embedded chalk particles in the system became smaller and about 1 um (Figure 3C). Can pectinase digest chalk? Obviously not, but we found in the literature that pectin by pectinase digestion will produce β-galacturonic acid, is an acidic substance [23,24]. At the same time, we tested the pH value of PC–DOX before and after mixing with pectinase (Table 2), and found that the pH value gradually decreased with time (Figure 3B). This may explain why not only pectin was decomposed, but also chalk particles became smaller after the pectinase effect. However, this acidity was limited, not enough to digest chalk completely and release DOX completely. On the other hand, we thought that nano- and microdrugs could also be swallowed by cells, so we tested the uptake of PC–DOX by tumor cells after pectinase digestion. Through fluorescence quantification, 77% of the fluorescence compared to that in the DOX-treated group could be detected (Figure 3D). This shows that although only a small amount of DOX was released from pectinase-treated PC–DOX, the DOX adsorbed on chalk was also taken up by cells due to the enzymatic hydrolysis of acid production, which made the chalk particles smaller and facilitated the endocytosis of tumor cells. In general, although the final DOX intake could not be compared with that in the pure DOX treatment group, it was also a high dose compared to other groups.

### 2.4. Distribution of PC–DOX in the GI Tract

**Figure 4:**
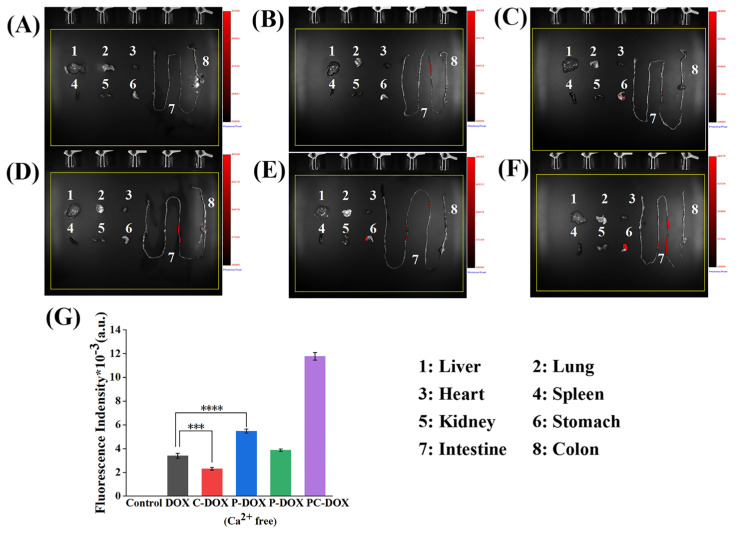
The organs of different groups: 1: liver, 2: lung, 3: heart, 4: spleen, 5: kidney, 6: stomach, 7: intestine, 8: colon. (**A**) Pectin–Ca^2+^ gels for the control group, (**B**) free DOX group, (**C**) C–DOX group, (**D**) P–DOX (Ca^2+^ free) group, (**E**) P–DOX group, and (**F**) PC–DOX group were pictured by the Small Animal Live Imaging System. (**G**) Summary of fluorescence intensity for each group *** *p* < 0.001, **** *p* < 0.0001 (*n* = 3). PC–DOX had the highest fluorescence content in all of the groups, which means the PC–DOX had an excellent ability to locate the colon.

Finally, we tested the drug distribution of PC–DOX in mice. After fasting for 12 h, we fed different groups of mice with different preparations by gavage. After 3 h, the mice were sacrificed, and the organs, including the stomach and intestines, of the mice were taken out. Fluorescence imaging was used and quantified. As shown in Figure 4, the fluorescence intensity of the PC–DOX group was the highest. However, our aim was to do colon-specific administration, but there was no fluorescence signal in the colon of mice in any of the treatment groups. This was because our formulation was designed according to the human intestinal rhythm, such as food residence time in the stomach of generally 2–6 h, so we chose the time of 2 h to simulate drug release in gastric juice [25,26]. Similarly, food retention time in the small intestine is generally 6–12 h, so, we chose the time of 6 h to simulate the release of small intestine. However, mice after eating defecate after 2–6 h [27]. We tried longer in vivo drug distribution (data not shown), when the food has been all excreted, so finally chose 3 h in vivo distribution experiments. Although the drug had not yet reached the colon, it can be seen in the small intestine that the PC–DOX group had the highest drug distribution, which also verified that PC–DOX could best resist the destructive effect of gastric acid. From this result, we could also infer that the colon will have the highest drug delivery efficiency.

## 3. Conclusions

In summary, in order to improve the delivery efficiency of the colon site of oral administration and prevent the penetration of drugs in the stomach, this study set up double-layer protection for DOX, the adsorption of chalk and the cross-linking encapsulation of pectin gel. The results showed that the rationally designed PC–DOX group had the lowest release in the stomach and small intestine compared with that in multiple control groups and could also release most in the simulated colon environment. On the one hand, the enzymatic hydrolysis of pectin could release free DOX. On the other hand, the acid produced by hydrolysis could decompose a part of chalk and further promote the release of DOX. In vivo distribution experiments also showed that the PC–DOX group could maintain the highest distribution in the intestine. The above results show that the drug delivery system designed in this study has the potential to be applied in colon diseases, including colon cancer. Next, we will establish a mouse orthotopic colon tumor model to further verify the anti-tumor effect of PC–DOX series in vivo.

## 4. Materials and Methods

### 4.1. Materials

Doxorubicin hydrochloride (DOX, 98%), pectin (Galacturonic acid ≥ 74.0%), pectinase from *Aspergillus niger*, Calcium chlorideanhydrous (CaCl_2_), and other chemicals and reagents that are not otherwise stated were supplied by Admas (CHN). Chalk was purchased from Xinchengkuangchanpin (CHN). Dulbecco’s modified Eagle’s medium (DMEM) and fetal bovine serum (FBS) were supplied by Gibco Life Technologies (Grand Island, NY, USA). Trypsin and penicillin–streptomycin were supplied by Beyotime Biotechnology (CHN).

All animal experiments were conducted under the guidelines approved by the Institutional Animal Care and Use Committee of Changzhou University. Female BALB/c mice (6–8 weeks, 18–22 g) were purchased from Shanghai Experimental Animal Center (Shanghai, China).

### 4.2. Preparation and Characterization of PC–DOX

At first, we prepared C–DOX by encapsulating DOX diffusion into chalk. According to previous reports, the loading technique (diffusion) consisted of continuous stirring of the mixture of a CaCO_3_ suspension and a DOX solution for 24 h [28].

Therefore, 500 μL of 1 mg/mL DOX solution were placed in a 5 mg/mL chalk suspension at the ratio of 1:1, 1:2, 1:5, 1:10, 1:15, and 1:20 (*w*:*w*), then, water was added to the mixture until the total volume was 5 mL (DOX, 100 μg/mL). The mixture had to be stirred for 12 h and centrifuged at 8000 rpm for 3 min after mixing. After centrifugation, the supernatant was measured for fluorescence intensity, and the concentration of residual DOX in the supernatant was determined, which could be used to calculate encapsulation efficiency.

(1) $$\mathrm{EE}=\frac{\mathrm{Initial}\;\mathrm{concentration}-\mathrm{Residual}\;\mathrm{concentration}}{\mathrm{Initial}\;\mathrm{concentration}}\times 100\%\;$$

Secondly, we chose the external gelation method for preparing pectin–Ca^2+^ gels. The external gelation method is also known as the diffusion setting method, which allows Ca^2+^ to freely diffuse into the pectin [22]. Pectin of different concentrations (1%, 2%, 3%, 5%, and 6%, *w*:*v*) was prepared before the experiment, a 10% CaCl_2_ solution was added to the pectin to form a gel (pectin: 10%, CaCl_2_ = 2.5:1, *v*:*v*), a 5 mg/mL chalk suspension was added to the gel, the mixture was stirred and mixed well, the gel phenomena was observed carefully between the perpendicular Eppendorf tubes and the ones that flipped immediately after the formation of the gel, and the optimal concentration of pectin-Ca^2+^ gel was ultimately determined.

At last, a 10% CaCl_2_ solution was added to 3% pectin to produce the gel (pectin: 10% CaCl_2_ = 2.5:1, *v*:*v*), the 10 mg/mL C–DOX suspension prepared by the previous method was added to the gel, stirred, and mixed well to obtain the final product PC–DOX. After the formation of the gel, it was necessary to flip the Eppendorf tubes in order to compare the phenomena between the different groups.

The morphology of the samples, including chalk, C–DOX, pectin, and pectin-Ca^2+^, was characterized using a scanning electron microscope (SEM).

### 4.3. In Vitro Drug Release Experiments

Preparation of simulated gastric fluid (SGF) with pH 1.2: About 800 mL of distilled water and 10 g of pepsin were added to 9 mL of concentrated hydrochloric acid, and the pH was adjusted to 1.2 by using 0.1 M HCl or 0.1 M NaOH [29]. We set the parameters of the transdermal diffusion to 37 °C at 100 rpm in order to simulate the drug release experiment in the stomach. The samples prepared in advance in 2.2 were packed into a dialysis bag (3.5 kDa), the dialysis bag was placed in a vial containing 1 L SGF at 0, 5, 10, 15, 30, 45, 60, 90, and 120 min. We needed to take out the release solution to measure the DOX fluorescence content at that point via using an FS5 spectrofluorometer (FS5, Edinburgh Instruments, UK), and then add the pre–warmed SGF, which was of the same volume as the amount taken out, to make up the volume of the released solution. At the end, according to the following formula, it was simple to calculate the cumulative drug release rate Q_n_.

Preparation of simulated intestinal fluid (SIF) with pH 6.8: Accurately weighed 6.8 g of KH_2_PO_4_ was dissolved in 500 mL of water, and the pH was adjusted to 6.8 with a 0.1 M NaOH solution. Then, 10 g of trypsin was added to water to dissolve. After the two solutions were mixed, we added water until 1000 mL [29]. The following steps were the same as in simulating the release in the stomach except the points of taking out the release solution were changed to 0, 0.5, 1, 2, 4, 6 h.

(2) $$Q_{n}=\frac{C_{n}\times V+\sum_{i=1}^{n-1}C_{i}\times V_{i}}{W}\times 100\%\;$$

Q_n_: cumulative release rate of the nth drug; C_n_: concentration of release solution removed for the nth time, μg/mL; V: total volume, mL; C_i_: concentration of the release solution removed for the ith time, μg/mL; V_i_: volume of release solution removed at ith time, mL; W: total mass of the drug, μg.

Q_G_ and Q_I_ represent the cumulative release rate in GI, and the remaining drugs that are not absorbed by GI will eventually enter the colon site to achieve colon targeting. Therefore, according to the release rate in the stomach and intestinal, we can finally calculate the colon-targeting efficiency through Equation (3).

(3) $$\mathrm{Colon}-\mathrm{targeting}\;\mathrm{efficiency}=\left(100-Q_{G}\right)\times \left(100-Q_{I}\right)\times 100\;\%$$

where Q_G_ is the release rate in the stomach and Q_I_ is the release rate in the intestine.

### 4.4. Pectin Enzymolysis

The preparation of PC–DOX that will be enzymatically hydrolyzed: 10% CaCl_2_ solution was added to 3% pectin to produce the gel (pectin: 10% CaCl_2_ = 2.5:1, *v*:*v*), after that, 10 mg/mL of C–DOX suspension was added to the gel, stirred, and mixed to obtain PC–DOX, which was the final product. A certain amount of 2% pectinase (pectin: pectinase = 5:1, *v*:*v*) was added to the PC–DOX previously prepared, the mixture was incubated in a 37 °C constant temperature shaker for 8 h, removed from the shaker, an in the supernatant that was obtained after low-speed centrifugation DOX fluorescence was measured content though an FS5 spectrofluorometer (FS5, Edinburgh Instruments, Livingston, UK).

The morphology of PC–DOX that was enzymatically hydrolyzed was characterized using a scanning electron microscope (SEM).

### 4.5. Cell Uptake

CT26 (mouse colon cancer cells) was cultured in DMEM containing 10% (*v*:*v*) FBS (Gibco Life Technologies, USA) and 1% (*v*:*v*) penicillin–streptomycin (Beyotime Biotechnology, Shanghai, China). Cells were incubated at 37 °C in a humidified incubator with 5% CO_2_ (Thermo Forma 311, Thermo Scientific, Waltham, MA, USA).

CT26 cells in the logarithmic growth phase were rinsed with PBS, digested with trypsin, then centrifuged at 1500 rpm for 3 min to pellet the cells. The cells were then counted using a cell counter, and seeded into a 24-well plate at a density of 15 × 105 cells·well-1. At the end, the cells were cultured for about 16–18 h.

The preparation and enzymolysis of PC–DOX could refer to the steps in Section 4.4. After enzymolysis, PC–DOX was diluted with DMEM (DOX, 2.5 μg/mL), 1 mL of diluted sample was incubated with the cells for 4 h and cultured at 37 °C in a 5% CO_2_ incubator. After that, the cells were washed with PBS and imaged using a Nikon eclipse Ti-S inverted fluorescence microscope (Ti-S, Nikon Corporation, Tokyo, Japan).

Finally, the cells were digested with trypsin, then centrifuged at 1500 rpm for 3 min to pellet the cells. Using the DMEM medium to resuspend the cells and using a flow cytometer (BD C6plus, Waltham, MA, USA), we quantified the fluorescence intensity in cells.

### 4.6. In Vivo Drug Release Experiments

Female BALB/c nude mice weighing about 20 ± 2 g were selected for oral administration. The standard dosage for oral administration was 0.1–0.2 mL/10 g, in other words, these mice could be gavaged 0.2–0.4 mL of the samples.

These mice were divided into six groups randomly as follows: pectin-Ca^2+^ gels for the control group, free DOX group, C–DOX group, P–DOX (Ca^2+^-free) group, P–DOX group, and PC–DOX group.

Before the experiment, the mice were forbidden from eating and allowed to drink water for 12 h. The mice were administered the drugs orally by gavaging and sacrificed after 3 h of gavage. After that, their internal organs (heart, liver, spleen, lung, kidney, stomach, and intestines) were taken in vitro and pictured by the Small Animal Live Imaging System (Tanon X6, Shanghai, China).

### 4.7. Statistical Analysis

All statistical analyses were performed using GraphPad Prism software version 9.1.0 (Harvey Motulsky, CA, USA). The results of experiments were expressed as the mean ± standard deviation (SD, *n* = 3). Statistical comparisons were performed by one-way or two-way analysis of variance (ANOVA) for multiple groups.

## Figures and Tables

**Table 1:** The table of colon-targeting efficiency.

	Stomach (%)	Intestinal (%)	Colon-Targeting Efficiency (%)
DOX C–DOX P–DOX (Ca^2+^ free)	84.58	91.40	1.33
	74.69	7.28	23.47
	65.67	68.69	10.75
P–DOX	54.48	55.19	20.42
PC–DOX	25.93	7.80	68.29

**Table 2:** Different pH of reactants used to prepare and enzymatically hydrolyze PC–DOX.

	pH Value
3% pectin	3.55
3% pectin adjusted to neutral	6.99
2% pectinase	5.4
10% CaCl_2_	9.0
Pectin-Ca^2+^ gel	7.99
